# First comparative genomics analysis of *Corynebacterium auriscanis*

**DOI:** 10.1590/0074-02760240156

**Published:** 2024-10-25

**Authors:** Ana Lua de Oliveira Vinhal, Max Roberto Batista de Araújo, Evandro Bento Rodrigues, Diogo Luiz de Carvalho Castro, Carine Rodrigues Pereira, Dircéia Aparecida Costa Custódio, Elaine Maria Seles Dorneles, Flávia Figueira Aburjaile, Bertram Brenig, Vasco Azevedo, Marcus Vinicius Canário Viana

**Affiliations:** 1 Universidade Federal de Minas Gerais, Departamento de Genética, Ecologia e Evolução, Belo Horizonte, MG, Brasil; 2 Instituto Hermes Pardini-Grupo Fleury, Microbiologia, Núcleo de Operações Técnicas, Vespasiano, MG, Brasil; 3 Universidade Federal de Lavras, Faculdade de Zootecnia e Medicina Veterinária, Departamento de Medicina Veterinária, Lavras, MG, Brasil; 4 Universidade Federal de Minas Gerais, Escola de Veterinária, Belo Horizonte, MG, Brasil; 5 University of Göttingen, Institute of Veterinary Medicine, Göttingen, Germany

**Keywords:** Corynebacterium, comparative genomics, zoonosis

## Abstract

**BACKGROUND:**

*Corynebacterium auriscanis* is a bacterial species frequently isolated from dogs with external otitis or dermatitis and a zoonotic pathogen transmitted by dog bite. It is considered an opportunistic pathogen, but its pathogenicity mechanisms are poorly studied. Comparative genomics can identify virulence and niche factors that could contribute to understanding its lifestyle.

**OBJECTIVES:**

The objectives of this project was to compare genomes of *C. auriscanis* to identify genes related to its virulence and lifestyle.

**METHODS:**

The genome of strain 32 was sequenced using Illumina HiSeq 2500 (Illumina, CA, USA) and assembled using Unicycler. The two other non-redundant genomes from the same species available in GenBank were included in the analysis. All genomes were annotated and checked for taxonomy, assembly quality, mobile elements, CRISPR-Cas systems, and virulence and antimicrobial resistance genes. The virulence genes in the three genomes were compared to the ones from other pathogens commonly isolated with *C. auriscanis*.

**FINDINGS:**

The species has 42 virulence factors that can be classified as niche factors, due to the absence of true virulence factors found in primary pathogens. The gene *rbpA* could confer basal levels of resistance to rifampin.

**MAIN CONCLUSIONS:**

The absence of true virulence factors in the three genomes suggests *C. auriscanis* has an opportunistic pathogen lifestyle.

*Corynebacterium auriscanis* is a Gram-positive bacterium species first isolated from dogs with external otitis.^1^ It is frequently isolated in canine otitis with other bacteria as *Staphylococcus intermedius* and *Streptococcus canis*, and the yeast *Malassezia pachydermatis.* ^2^ Similarly, it can be found with other known pathogens in cases of canine dermatitis.^3^ This bacterium also exhibits zoonotic potential, having been isolated from infected wounds caused by dog bites.^3^ ^,^ ^4^ Due to co-isolation with other species, *C. auriscanis* is considered an opportunistic pathogen, although it was also isolated alone from lesions in dogs^3^ and in a human.^4^

Genomic data could reveal the pathogenic potential of the species, as well as other characteristics of veterinary and medical relevance, such as antimicrobial resistance genes and sequence type.^5^ As of January 2024 only four genomes of the species were publicly available in GenBank (https://www.ncbi.nlm.nih.gov/datasets/genome/?taxon=99807), including the one sequenced in this study.

To better understand the molecular mechanisms involved in the virulence and lifestyle of the species, we sequenced the genome of strain 32, isolated from a dog ear with otitis in Mexico. We then compared this genome to the other publicly available genomes. This represents the first comparative genomic analysis of *C. auriscanis*.

## MATERIALS AND METHODS

*Genome sequencing, assembly and annotation* - The strain 32 was grown overnight in brain heart infusion (BHI) broth supplement with Tween 80 under aeration at 37ºC. Genomic DNA was extracted using the Wizard Genomic DNA Purification Kit (Promega) following the standard protocol. DNA was sequenced using ThruPLEX DNA-Seq Kit (Takara) and Illumina HiSeq 2500 (Illumina, San Diego, CA, USA) with 150b paired-end reads. Sequencing read quality was assessed with FastQC v. 0.11.9.^6^ A de novo assembly was performed using Unicycler v. 0.4.8.^7^ Assembly quality was evaluated using CheckM2 v. 1.0.2^8^ for completeness and contamination, GUNC v. 1.0.6^9^ for chimeric contigs, and QUAST v. 5.1.0rc1^10^ for fragmentation. Taxonomic classification was performed using GTDB-Tk v. 2.2.6.^11^ and Type Strain Genome Server (TYGS).^12^ The presence of plasmids was investigated using MOB-suite v. 3.1.8.^13^ Contigs with less than 200 bp were removed using Seqkit v. 2.1.0.^14^ The genome was annotated using NCBI Prokaryotic Genome Annotation Pipeline (NCBI’s PGAP).^15^

*Genome characterisation* - For further comparison and characterisation, two additional strains were retrieved from GenBank, using the RefSeq database^16^ to ensure consistent genomic annotation. The Type strain, represented by DSM44609 (GCF_030408435.1) and CIP106629 (GCF_000767255.1), was isolated from a dog with an ear infection in the United Kingdom (SAMN13404511). bin-22 (GCF_029984515.1) is a metagenome-assembled genome isolated from a cat’s anal gland secretion from USA (SAMN34353469). CIP106629 was not used, as it is same strain as DSM44609. Completeness and contamination of all *C. auriscanis* genomes were verified. Virulence or niche factors^17^ ^,^ ^18^ were predicted using VFanalyzer from VFDB website (http://www.mgc.ac.cn/cgi-bin/VFs/v5/main.cgi?func=VFanalyzer)^19^ and PanViTa v1.1.5^20^ with the Virulence Factor Database (VFDB).^21^ The PanViTa script (panvita.py) was modified to utilise the full VFDB dataset before installation using the command line: “sed -i ‘s/VFDB_setA_pro/VFDB_setB_pro/’ panvita.py”. Antimicrobial resistance genes were predicted using PanViTa with the Comprehensive Antibiotic Resistance Database (CARD).^22^ Prophages and CRISPR-Cas systems were predicted using PHASTER^23^ and CRISPRCasFinder v. 1.1.2,^24^ respectively. Genomic islands (GIs) were predicted in the type strain DSM44609 using GIPSy v1.1.3^25^ with *C. glutamicum* ATCC13032 (GCF_002847405.1) as the non-pathogenic reference strain. In GIPSy, identified GIs are classified as Metabolic Islands, Pathogenicity Islands, Resistance Islands, or Symbiotic Islands based on the proportion of BLASTp hits against its databases of virulence, resistance, metabolism, and symbiotic genes. Hits with identity values ≥ 70% were considered.

*Comparative genomics* - A circular map comparing DSM44609 to strains 32 and bin-22 was built using BRIG v. 0.95.^26^ Average Nucleotide Identity (ANI) values were calculated using pyani v. 0.2.12^27^ with the BLASTn algorithm. The gene repertoire of the strains was identified using PPanGGOLiN v. 2.0.5.^28^ Functional annotation using eggNOG-mapper v. 2.1.11 with eggNOG database v. 5.0.2.^29^ The investigation of virulence factors and mechanisms was performed using VFanalyzer and PanViTa. The three *C. auriscanis* genomes were compared to: (i) the pathogenic and zoonotic species of the same genus *C. ulcerans*, (ii) the non-pathogenic strain from the same genus *C. glutamicum*, and (iii) the species that are isolated alongside *C. auriscanis* from otitis *S. canis* and *S. pseudintermedius*.

## RESULTS

The genome of *C. auriscanis* 32 was assembled as a draft with 46 contigs. Completeness and contamination were estimated at 99.81 and 0.16%, respectively. The genome was deposited in GenBank under BioProject PRJNA764731 (https://www.ncbi.nlm.nih.gov/bioproject/PRJNA764731/) with assembly accession numbers GCA_026236545.1 (GenBank) and GCF_026236545.1 (RefSeq) (Table).

**TABLE:** Metadata and genomic features of *Corynebacterium auriscanis* strains

Strain	32	bin_22	DSM44609
Host	Dog	Cat	Dog
Country	Mexico	USA	United Kingdom
Isolation source	Ear infection	Anal gland secretions	Ear infection
Accession	GCF_026236545.1	GCF_029984515.1	GCF_030408435.1
Contigs	46	52	1
Size	2,507,534	2,372,762	2,599,172
GC (%)	58.8	58.64	58.49
N50	226,683	74,239	2,599,172
Completeness (%)	99.81	94.83	99.91
Contamination (%)	0.16	0.08	0.01
Taxonomy (GTDB-Tk)	*C. auriscanis*	*C. auriscanis*	*C. auriscanis*
Taxonomy (TYGS)	*C. auriscanis*	*C. auriscanis*	*C. auriscanis*
Plasmid	None	None	None
Complete prophages	0	0	0
Incomplete prophages	0	3	1
CDS	2,096	1,975	2,140
Pseudogenes	47	40	46
tRNA	50	30	50
rRNA	3	0	9
CRISPR arrays	0	0	1
CRISPR-Cas system	-	-	Type I-E
Antimicrobial resistance genes	2	2	2
Virulence factors	38	38	40

CDS: coding sequence; CRISPR: clustered regularly interspaced short palindromic repeats; GC: guanine-cytosine; GTDB-Tk: genome taxonomy database toolkit; TYGS: type strain genome server.

Strains 32 and bin-22 were classified as *C. auriscanis* due to ANI > 95% (GTDB-Tk) and digital DNA-DNA Hybridisation > 70% (TYGS) when compared to the type strain CIP106629. DSM44609 is the same strain as CIP106629 (https://www.dsmz.de/collection/catalogue/details/culture/DSM-44609) [Supplementary data (Tables I-II)]. All three strains possess the same two predicted resistance genes to rifampin (*rpoB2* and *rbpA*) [Table, Supplementary data (Table III)]. Three incomplete prophages were predicted in strain bin-22, with one incomplete prophage in strain DSM44609. All classified as different prophages, none were identified in strain 32. Only DSM44609 had a CRISPR-Cas system, classified as Type I-E [Supplementary data (Tables IV-V)]. A total of 27 GIs harbouring 617 genes were predicted in strain DSM44609. Using a 70% cut-off (consistent with PanViTa) on the BLASTp results of GIPSy, we identified one gene across the GIs involved in virulence (*glnA1*), 28 genes in metabolism, and none associated with antimicrobial resistance or symbiotic lifestyle (Figure) [Supplementary data (Tables VI-VII)].

ANI between the genomes ranged from 97.18 to 97.64%. A total of 2,266 protein-coding genes were identified across the three genomes, categorised into 2,013 persistent (88.83%), 142 shell (6.27%) and 111 cloud (4.90%) genes. Regarding exclusively present genes, strain 32 has 111, bin-22 has 62 (21 from a prophage), and strain DSM44609 has 142 (48 from a prophage) genes [Supplementary data (Tables VIII-X)].

In *C. auriscanis* a total of 42 niche or virulence factors were predicted, with 10 exclusively predicted by PanViTa, 19 by VFanalyzer, and 11 by both tools. Five of these genes are involved in adhesion (*groEL2*, *sapD*, *spaD*, *srtC* and *tufa*). In the metabolism category, the genes are associated with leucine and glutamine synthesis (*leuD* and *glnA1*); uptake of heme (Rv0207c), iron (*ciuABCD*, *fagAB*, GBAA_RS25985, *hmuTUV*, *mbtI*), and copper (*ctpV*); lipid and fatty acid metabolism (*icl*, *panC*, *panD*); trehalose transport (*sugC*); amino acids and purine metabolism (*lysA* and *proC*); and protein degradation (*mpa*, *pafA*). Other genes are involved in oxidative stress and low oxygen response (*katG*, *regX3*) and acidic stress (*ureB*, *ureG*). Genes involved in gene regulation are two alternative sigma factors (*sigA* and *sigH*), a transcription activator of *sigA* (*whiB3*), a stress-responsive two-component system (*mprA*) and the diphtheria toxin repressor (*dtxR*). Two genes are part of secretion systems (*secA2* and *espI*), one is involved in biosynthesis of glycopeptidolipids (*rmlA*), other in immune modulation (*nkd*) and other in immune evasion (*pgi*). Fifteen of the 42 genes are in GIs [Supplementary data (Tables XI-XII)].

Among the three *C. auriscanis* genomes, strain DSM44609 has 40 niche or virulence factors, with *pgi* and *espI* being exclusive genes. Strains 32 and bin-22 each have 38 genes. Strain 32 is distinguished by the absence of *sapD* and *fagA* and the presence of *ureB* and *ureG*. No toxin-encoding gene was found in any of the three genomes. Out of the total 42 niche or virulence factors, 18 are not present in the free-living *C. glutamicum* ATCC13032. From these 18, nine are in GIs and 17 are present in the pathogen *C. ulcerans* 4290 (Figure) [Supplementary data (Table XI)]. Toxins (*C. ulcerans*, *S. canis* and *S. pseudointermedius*) and an invasion protein (*S. pseudointermedius*) we identified only in pathogenic species [Supplementary data (Table XI)].

## DISCUSSION

*Corynebacterium auriscanis* is suggested to be an opportunistic pathogen due to its frequent isolation alongside other species causing external otitis or dermatitis in dogs, and occasionally as the sole agent in dogs and humans with infected wounds caused by dog bites.^2^ ^,^ ^3^ Our objective was to identify virulence factors that could explain its lifestyle and conduct the first comparative genomic study of this species.

Strain bin-22 exhibits a smaller genome size and fewer protein coding sequences (CDS) and tRNA (Table), probably due to errors in the binning process, during contig attribution from the original metagenome sample. Within the three genomes, ANI values of approximately 97% and the classification of 88.83% of genes as persistent [Supplementary data (Tables VIII-X)] indicate that the genomes are not clonal. The different prophages across the genomes might contribute to the difference (Figure) [Supplementary data (Table XI)]. A more comprehensive analysis of additional genomes would be beneficial for a better understanding of the species’ genetic diversity.

**Figure:**
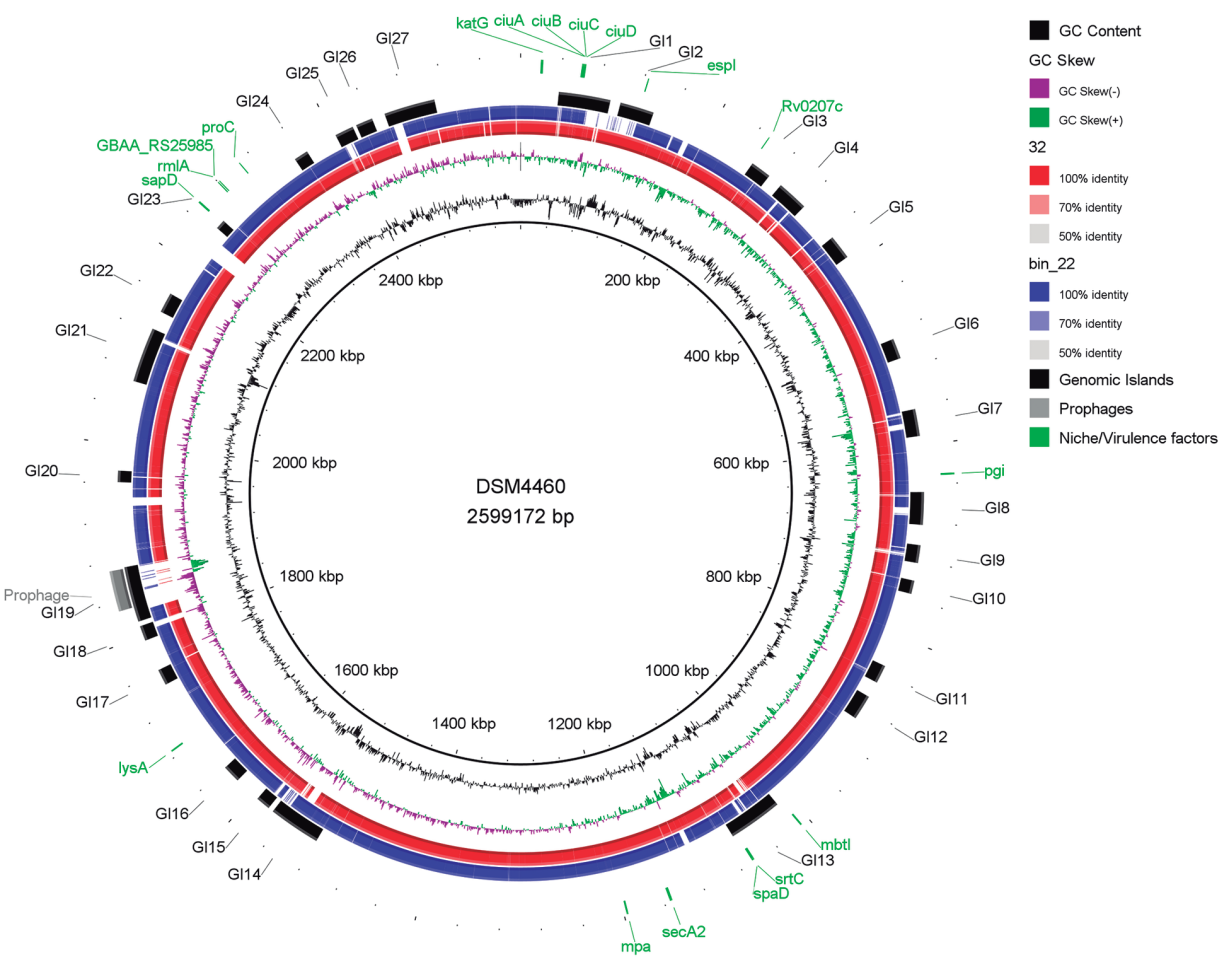
Circular map of *Corynebacterium auriscanis* DSM44609. The 18 niche or virulence factors shown are not shared with *C. glutamicum* ATCC13032. GC: guanine-cytosine; GI: genomic island.

CRISPR-Cas systems are adaptive immune systems of bacteria that protect against exogenous DNA, such as phages and plasmids.^30^ We identified a CRISPR-Cas system Type I-E in *C. auriscanis* strain DSM44609 (*cas*3, *cse*1, *cse*2, *cas*7, *cas*5, *cas*6, *cas*1, *cas*2) [Supplementary data (Table V)]. This type is well known in *Corynebacterium* species such as *C. diphtheria*,^31^ *C. pseudotuberculosis*,^32^ *C. rouxii* ^33^ *and C. striatum.* ^34^

PanViTa identified two genes from the CARD database within the three *C. auriscanis* genomes that could potentially confer rifampin resistance: *rpoB2* and *rbpA* [Supplementary data (Table III)]. The gene *rpoB2* in the CARD database is a rifampin-resistant paralog of the *rpoB* gene, which encodes the β subunit of bacterial RNA polymerase (RNAP), as described in *Nocardia farcinica*.^35^ The identification of *rpoB2* in the three genomes of *C. auriscanis* is a false positive. Given that these genomes only contain a single copy of *rpoB*, the *rpoB2* sequence from the CARD database, with 71.8% identity, was likely the best alignment hit (PPanGGoLiN results in Supplementary data). The *rbpA* gene encodes an RNA polymerase-binding protein commonly found in actinomycetes. It is part of the σ^R^ regulon and confers basal levels of resistance to rifampin by protecting the product of *rpoB.* ^36^

Genomic islands are horizontally acquired regions that can contribute to adaptations to different environments and bacterial evolution.^37^ Using *C. glutamicum* ATCC13032 as a non-pathogenic reference genome, we identified 27 GIs within the complete genome of *C. auriscanis* DSM44609. Many of these GIs are shared with strains 32 and bin-22 (Figure) [Supplementary data (Table VI)]. Since we compared *C. auriscanis* to the free-living bacterium *C. glutamicum*, the identified GIs may contain exclusive genes related to its commensal lifestyle.^38^

The discrepancies in the prediction of virulence factors by PanViTa and VFanalyzer, despite their reliance on the same VFDB database, are likely attributable to the distinct algorithms they employ. PanViTa aligns protein sequences from query and database using DIAMOND-BLASTp and applies a filter of 70% identity and 70% coverage.^20^ VFanalyzer identifies orthologous genes within the query genome and the reference genomes from the database to mitigate false positives due to paralogs. It subsequently conducts iterative sequence similarity searches within hierarchical datasets encompassing other genera to identify atypical or strain-specific genes, and ultimately performs a refinement process that incorporates the genome context.^19^

Within the three *C. auriscanis* genomes, we identified 42 virulence or niche factors. None of these factors qualify as true virulence factors. However, we did uncover toxins and an invasin in the three primary pathogens *C. ulcerans*, *S. canis* and *S. pseudointermedius* [Supplementary data (Table XI)]. The term “niche factor” was coined to describe components involved in the colonisation of distinct ecological niches, rather than specific interactions between pathogens and hosts. These factors are prevalent in bacteria with diverse lifestyles. Their role in pathogenicity depends on their combination with true virulence factors and specific environments. Examples of niche factors include adhesins, surface-anchored enzymes, cell envelope components, and antimicrobial resistance.^17^ ^,^ ^18^ True virulence factors enable pathogens to gain access or survive in body sites not typically colonised, cause damage to the body, and lead to disease symptoms due to the dysregulation of the immune system or neurological responses. Examples of such factors include internalins, invasins, cytolytic or hemolytic toxins, superantigens, and neurotoxins.^17^ In *Corynebacterium* known true virulence factors are the toxin encoding genes *tox* (diphtheriae toxin) and *pld* (phospholipase D).^18^ Given these distinctions, the 42 virulence or niche factors identified in the three *C. auriscanis* genomes can be classified as niche factors rather than true virulence factors.

Eighteen of the 42 niche genes of *C. auriscanis* are absent in the free-living *C. glutamicum*, and 17 of these 18 are shared with the pathogen *C. ulcerans* [Supplementary data (Table XI)]. These 17 genes likely represent adaptations to a host-associated lifestyle. Some of these adaptations may directly involve bacterial-host interactions, such as proteins involved in adhesion (*sapD*, *spaD* and *srtC*), immune system evasion (*ndk* and *pgi*), secretion systems (*espI* and *secA2*) and oxidative stress resistance (*katG*), while others are involved in nutrient uptake or metabolism.

In conclusion, the absence of true virulence factors in the three genomes of *C. auriscanis* suggests that the species is not a primary pathogen but rather an opportunistic one, thriving only when associated with pathogenic bacteria or in damaged tissues. However, further analysis of additional genomes is necessary to evaluate the genetic diversity of the species, which could include genomic islands or virulence factors that may confer strain-specific pathogenic potential.
